# Algorithms for Lightweight Key Exchange †

**DOI:** 10.3390/s17071517

**Published:** 2017-06-27

**Authors:** Rafael Alvarez, Cándido Caballero-Gil, Juan Santonja, Antonio Zamora

**Affiliations:** 1 Department of Computer Science and Artificial Intelligence, University of Alicante, 03690 Alicante, Spain; jsl2@alu.ua.es (J.S.); zamora@dccia.ua.es (A.Z.); 2 Department of Computer Engineering and Systems, University of La Laguna, 38206 Tenerife, Spain; ccabgil@ull.es

**Keywords:** public-key, key exchange, lightweight cryptography, elliptic curve, security, privacy, sensor networks, internet of things

## Abstract

Public-key cryptography is too slow for general purpose encryption, with most applications limiting its use as much as possible. Some secure protocols, especially those that enable forward secrecy, make a much heavier use of public-key cryptography, increasing the demand for lightweight cryptosystems that can be implemented in low powered or mobile devices. This performance requirements are even more significant in critical infrastructure and emergency scenarios where peer-to-peer networks are deployed for increased availability and resiliency. We benchmark several public-key key-exchange algorithms, determining those that are better for the requirements of critical infrastructure and emergency applications and propose a security framework based on these algorithms and study its application to decentralized node or sensor networks.

## 1. Introduction

Public-key cryptography is very demanding in terms of computing power but unavoidable in most modern secure applications, even those intended for low power or mobile devices. For this reason, public-key cryptography has been considered too slow for general purpose encryption, with applications employing public-key cryptography exclusively to securely share keys for the much faster standard symmetric-key cryptography (see [1,2]); limiting, in this way, the use of public-key cryptography as much as possible.

With the recent aim of perfect forward secrecy in many protocols, however, this model is no longer possible since these algorithms generate new key pairs and exchange secret keys per session and sometimes even per message (see [3,4,5]); therefore increasing the demand for lighter public-key cryptography, especially for peer-to-peer applications involving mobile wireless devices.

Moreover, secure communication protocols based on peer-to-peer and other types of *ad-hoc* networking are especially useful in critical infrastructure and emergency situations since they can enable improved coverage, resiliency, connectivity, security, anonymity and data privacy in these challenging applications.

The main contributions of this paper are:

- Performance analysis of several state-of-the-art public-key key-exchange cryptographic algorithms (see [6]) in order to find those that are most suitable for critical infrastructure and emergency applications, embedded and low power computing platforms or sensor networks. These primitives can be implemented successfully on most modern platforms with sufficient computing power such as standard ARM system on a chip (SoC) or similar environments, mobile devices and other computing nodes that can support wired or wireless communications.
- The design of a security framework employing the analyzed primitives, with the aim of adding security to a previously published peer-to-peer audio conferencing protocol over a hypercube topology (see [7]). This framework employs an authentication system inspired by traditional Public Key Infrastructure systems since it is meant for tightly controlled sensor or node networks and is based primarily on the recent FourQ elliptic curve [8,9] and well-known AES cryptosystems due to performance reasons, but other authentication methods and cryptographic primitives are also discussed.

The rest of the paper is organized as follows: Section 2 contains the state of the art analysis as well as performance results and insight of alternative key exchange protocols that could be used in certain scenarios, Section 3 presents our proposal for a security framework and Section 4 concludes the paper with some remarks and future research possibilities.

## 2. State of the Art Analysis

We describe in the following some public-key protocols that are commonly used for symmetric key agreement, with a special focus on performance.

### 2.1. RSA

Extremely popular, RSA is one of the first practical public-key cryptosystems (first published in 1977 by Ron Rivest, Adi Shamir and Leonard Adleman) and bases it security on the difficulty of factoring the product of two large prime numbers. Whether breaking RSA is as hard as the factoring problem is still an open question; although, if the public key is large enough, only someone with the knowledge of these two prime numbers can decode the message in a feasible way.

Unlike the other algorithms considered in this paper, RSA is a full public-key cryptosystem capable of directly supporting data encryption/decryption, key exchange and digital signature. As other public-key cryptosystems, RSA is too slow for general purpose data encryption, so it is mainly used to securely exchange symmetric keys and other small values. For more information see [2,10].

### 2.2. Diffie-Hellman

The Diffie-Hellman (DH) key exchange protocol was originally conceptualized by Ralph Merkle and designed by Whitfield Diffie and Martin Hellman in 1975. Its security is based on the discrete logarithm problem, which is considered unfeasible for groups of large enough order; unlike RSA, the DH key exchange protocol is not a full public-key cryptosystem and only enables for the exchange of a secret value that can be used for symmetric keys or other purposes, but does not support data encryption/decryption or digital signature directly.

The shared value is dependent on the asymmetric key pairs of both ends of the conversation, so new keys must be generated if a new secret is required. Protocols that achieve forward secrecy (see [3,4]) generate new key pairs for each session, discarding the old ones for each new session; the DH key exchange protocol is a common choice for these protocols since key pair generation is very fast. See [2] for additional information.

### 2.3. Elliptic Curve Diffie-Hellman

As in Section 2.2, the elliptic curve Diffie-Hellman (ECDH) key exchange protocol allows both ends of a conversation to establish a shared secret. It is an adaptation of the DH key exchange protocol but employing elliptic curve cryptography, which has some advantages in terms of key length and overall performance.

The ECDH protocol requires that both parties agree, prior to secure communication, on domain parameters and each party must generate a suitable key pair consisting of a private key *d* (a randomly selected integer in a certain interval) and a public key *Q* (a point in the curve). These public keys can either be static (and trusted via certificate) or ephemeral (usually referred to as ECDHE) that are temporary and not authenticated.

The National Institute for Standards and Technology (NIST) standardized some elliptic curves suitable for cryptography (see [11]) in different security levels. Elliptic curve cryptography is a vast and complex field, for more information see [12,13].

#### 2.3.1. Curve25519

Also referred to as X25519, Curve25519 is an ECDH key exchange protocol targeting the 128-bit security level and offering vastly improved performance compared to the traditional NIST elliptic curves. It was released by Daniel J. Bernstein in 2005 and constructed in such a way that it avoids many common problems in its implementation; eliminating, by default, many side channel attacks and issues with poor-quality random-number generators.

Besides pure performance, some suspicious aspects of the NIST P curves constants (there have been some concerns regarding their origin) have increased the popularity of Curve25519, making it the default for modern protocols like WhatsApp [5] and Signal [4], among others. For more detailed information regarding Curve25519, see [14,15,16].

#### 2.3.2. FourQ

FourQ is a high-performance elliptic curve that also targets the 128-bit security level. It is a fairly recent state-of-the-art design, being released by Microsoft Research in 2015 (see [8,9]); therefore, it is not used yet in standard or well-known protocols.

Its high performance stems from a four-dimensional decomposition minimizing the total number of elliptic curve group operations, extended twisted Edwards coordinates enabling the fastest known elliptic curve addition formulas, and extremely fast arithmetic modulo the Mersenne prime $p\;=\;2^{127}-1$. With this optimizations, FourQ is claimed to be between 4 to 5 times faster than NIST P-256 curve and 2 to 3 times faster than Curve25519.

### 2.4. Performance Analysis

In this section, we analyze the performance of several public-key algorithms in terms of key pair generation and secret exchange (key agreement). These two operations form the basis of most secure protocols that involve communication over insecure channels and are a suitable performance benchmark for such cases.

All benchmarks have been performed employing optimized implementations on an Intel Core i7-5930k CPU with 32 GB of RAM running Windows 10 Enterprise 64-bit, with all measurements computed as the average over 100 cycles of each specific operation and take into account a single side of the conversation (a single key pair generation and the calculation of a single party of the secret exchange).

In the case of RSA, the exchanged secret was 32 bytes (256 bit) long, and the exchange was performed by simple encryption with the recipient’s public key; in the rest of the algorithms, the exchanged secret was the expected length as per the algorithm’s design and parametrization.

We describe in Table 1 the key lengths required to achieve an equivalent security level with different algorithms (see [17]). We have targeted the 128-bit symmetric-equivalent security level, taking the following key lengths for each algorithm:

- RSA (3072 bit),
- Diffie-Hellman (3072 bit),
- Elliptic curve Diffie-Hellman (NIST P-256, which has a 256 bit key length),
- Curve25519 (key length is fixed at 256 bit),
- FourQ (key length is fixed at 256 bit).

#### 2.4.1. Key Pair Generation

**Figure 1:**
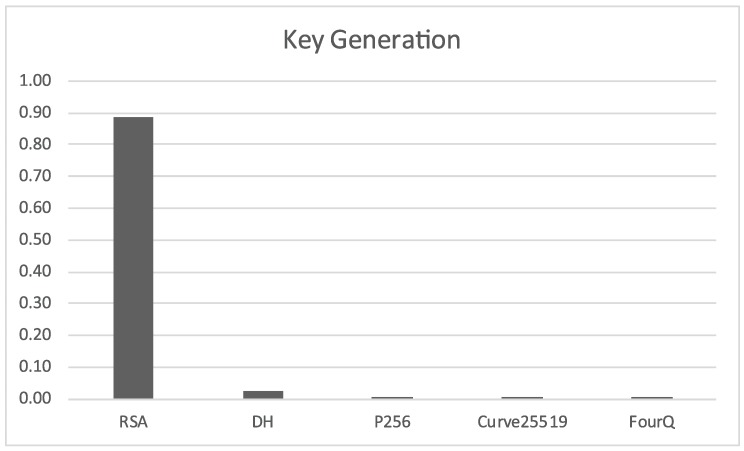
Time required *(in seconds)* for the generation of a single key pair.

We can see in Figure 1 that RSA is severely impacted by the long bit length required to maintain the security level target. Standard Diffie-Hellman is a bit slower than the elliptic curve variants, which all seem to be equivalent at this scale, but it is much quicker than RSA.

**Figure 2:**
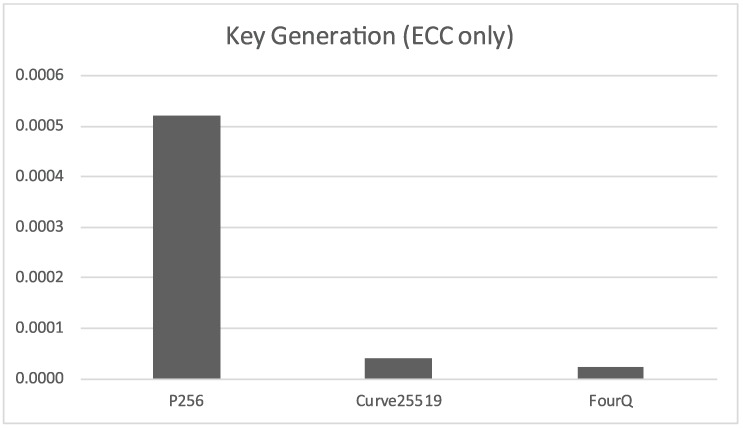
Time required *(in seconds)* for the generation of a single key pair *(elliptic curve algorithms only)*.

If we only take into account the elliptic curve Diffie-Hellman based algorithms, shown in Figure 2, we can see that the standard NIST P-256 curve is several times slower than Curve25519, and that FourQ is almost 2 times faster than Curve25519.

#### 2.4.2. Secret Key Exchange

**Figure 3:**
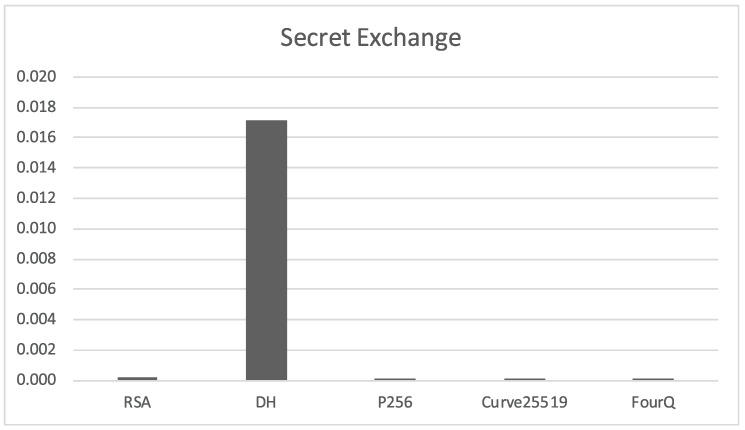
Time required *(in seconds)* for the exchange of a secret key on a single side of the communication.

In the case of the secret value exchange operation, the situation is reversed from the previous section with standard Diffie-Hellman being much slower than the rest of the algorithms and RSA being a bit slower than the elliptic curve variants (see Figure 3). It should be remarked that the overall times required for secret exchange are much less than the times required for key pair generation.

**Figure 4:**
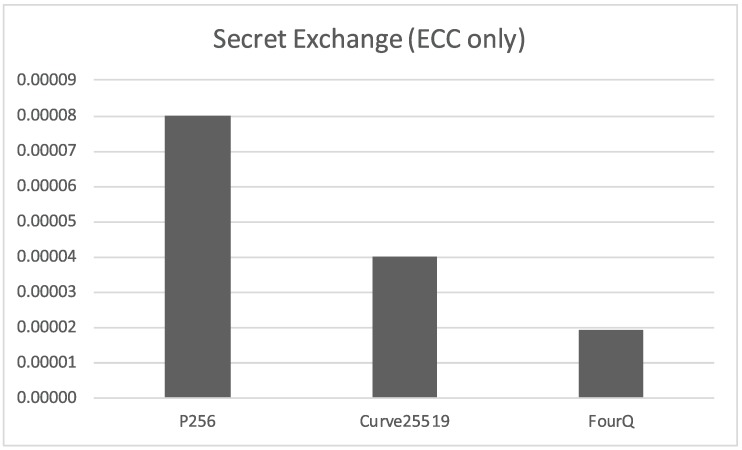
Time required *(in seconds)* for the exchange of a secret key on a single side of the communication *(elliptic curve algorithms only)*.

Moreover, focusing exclusively on the elliptic curve Diffie-Hellman variants (Figure 4), we find again that there are very tangible performance benefits with the Curve25519 and, especially, FourQ ECDH schemes.

### 2.5. Key Exchange Protocols

The direct application of key exchange algorithms based on asymmetric cryptography is vulnerable to Man-In-The-Middle (MITM) attacks; these can be prevented employing an authentication mechanism involving shared secret information with well-known techniques such as Certification Authorities (CA) and Public Key Infrastructure (PKI). This approach can be valid for friend-to-friend or previously registered node networks since the authorization phase is done as an offline initialization process, but can lead to performance and scalability issues in open peer-to-peer networks where there are no previous relationships between nodes. These situations require alternative side channels like ZRTP [18], where peers read aloud a short authentication string or a combination of authentication methods such as the 3AKEP protocol (see [19]).

## 3. Security Framework

In this section, we describe a security model based on the algorithms studied earlier in Section 2. This framework has been designed to add security to a decentralized or peer-to-peer network of low power autonomous computing nodes (such as smart phones) based on a hypercube topology with multiple applications in real-time streaming, like multi-party audio conferencing (see [7]), but it can also be adapted to other types of network topologies and node characteristics, since the security model described in the following is flexible enough to be suitable for a wide range of applications (see [20,21]).

All figures in this section follow a uniform notation representing the cryptographic methods and concepts employed in graphical form. In this way, public keys are shown as a green key icon, while private keys are shown in red and session keys in purple. Encrypted communications are represented as a continuous arrow line using the involved key color, while discontinuous arrow lines indicate secondary channels. Identification data together with a public key (certificates) are shown together as a paper to be signed; digital signatures are indicated as a red stamp.

### 3.1. Node Types

Regarding permissions or roles, we can distinguish four types of nodes: root, registered, registrar, and unregistered nodes; these are described in the following.

#### 3.1.1. Root Node

**Figure 5:**
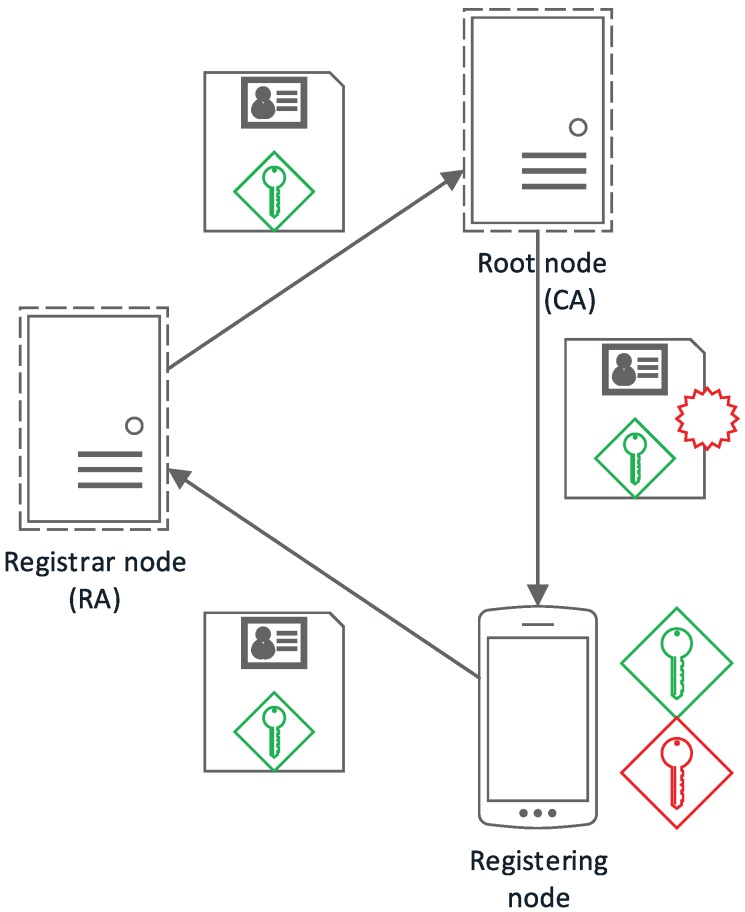
Node type relationship with traditional PKI roles.

The root node, $N_{root}$, would correspond to the CA on a conventional PKI setup (as shown in Figure 5); being in charge of legitimatizing the relationship between a public key and a node identifier. Initially, it is also the only Registration Authority (RA); although it is possible to delegate this responsibility to other registered nodes, registering new nodes that have been approved by other registered nodes. It is the first node in the network and acts as the network administrator.

The root node is convenient in applications where there is a set of previously registered nodes such as friend-to-friend networks or in tightly controlled sensor networks where authentication is performed in the initialization stage. In the case of open peer-to-peer networks, where a CA approach might not be adequate in terms of scalability or performance, the trust-adaptive approaches described in [22] are a further possibility, being a valuable option in scenarios where decentralization is critical.

#### 3.1.2. Registered Node

A registered node is a node that has been accepted and is a member of the network. In this scheme, in order to communicate and verify the identity of the rest of registered nodes, every registered node must have a public key that has been signed by $N_{root}$ and its associated private key, the public key of $N_{root}$, and an updated copy of the revocation list.

#### 3.1.3. Registrar Nodes

In a conventional PKI setup, registrar nodes would correspond to the Registration Authority. They take part in the node registration process, verifying the relationship between an unregistered node and its public key, as well as forwarding the public key to the root node so that it can be signed and accepted by the rest of the network.

Every registered node in the network can be, potentially, a registrar node, but only those chosen by $N_{root}$ will have this role in the network. This kind of node is optional and only applicable to those applications where delegated registration is required or makes sense.

#### 3.1.4. Unregistered Nodes

Any node that does not pertain to the network for any reason (expelled, not yet registered, etc.) is considered an unregistered node.

### 3.2. Network Setup

The initial setup of the network comes from a node initiating the process of establishing itself as the root node. After this process, there will be a network with a single node, that will grow after the registration of other nodes in the network.

This process consists of the generation of a public-private key pair and self-signing its public key and initializing the required repository to store the public key and the data associated to the nodes that will be added to the network.

#### 3.2.1. Node Addition

**Figure 6:**
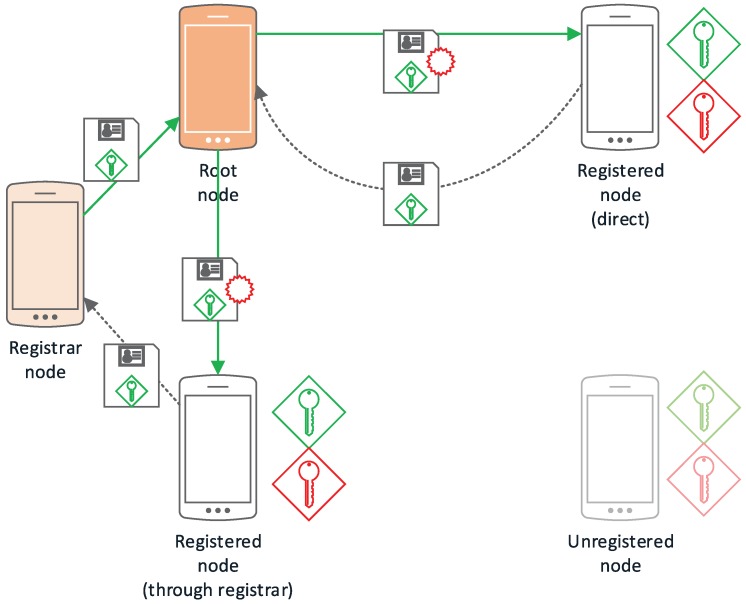
Network setup diagram.

As seen in Figure 6, a node ($N_{i}$) can request access to the network either by contacting the root node to be authenticated or contacting a node with RA permissions ($N_{RA}$).

In the case of requesting access directly to the root node, $N_{root}$ must check the identity of $N_{i}$ using an authentication method that provides an adequate security level for the network. In this process, $N_{root}$ verifies the relationship between the real identity and the public key of $N_{i}$, which has been self-generated. Then, $N_{root}$ signs the public key and returns it securely to $N_{i}$ together with its own public key. The exact mechanism for identity verification is application specific, so this framework is open-ended in this regard.

The channel used to perform this key exchange also depends on the security level required by the network but, for a medium-high security level, could consist of Near Field Communication (NFC) and/or Quick Response (QR) codes.

If the addition process is performed by a $N_{RA}$ then, after verifying the relationship between the real identity and the public key of $N_{i}$, it must transmit the registration request securely to $N_{root}$. This transmission can be instantaneous if both $N_{RA}$ and $N_{root}$ are online, or delayed until both nodes are available. In this case, $N_{RA}$ must store the registration request until it can be transmitted and, therefore, $N_{i}$ will not have complete access until $N_{root}$ successfully registers $N_{i}$ and propagates its credentials through the network. When $N_{root}$ receives the registration request, it can accept it automatically if it comes from a trusted registrar node or follow other protocol depending on the specific needs or characteristics of the network.

Regardless if the registration has been performed directly against $N_{root}$ or with a trusted $N_{RA}$, the root node must:

- Sign the public key of $N_{i}$.
- Assign $N_{i}$ an identifier in the network.
- Add the signed public key of $N_{i}$ to the repository.
- Propagate the new repository to all members of the network as they become available.

Once the new credentials are available to the network, then $N_{i}$ is accepted as a new member by the rest since they can check its signed public key against the repository. Propagation of the repository is performed via system messages within the underlying hypercube topology described in [7].

#### 3.2.2. Registration Delegation

As described previously, the root node can decide to automatically register any node that has been registered by other registrar nodes. This can be implemented simply by adding the identifiers of these $N_{RA}$ nodes to a list of trusted registrar nodes. This framework is open-ended regarding registrar trust implementation since it can be entirely application specific: in some cases it might be convenient to utilize real life relationships, node operator judgment or algorithmic methods such as trust scores, etc.

#### 3.2.3. Node Exclusion

In order to exclude a node from the network, the root node must add its identifier to the revocation repository and make sure this updated list is propagated correctly through the rest of the network. In this way, the nodes excluded via the revocation list will be ignored in future propagation of the network credentials repository.

### 3.3. Service Setup

In the hypercube protocol described in [7], a service corresponds to a real-time audio stream involving a set of nodes (speakers and listeners) in the network. In order to support multiple simultaneous conversations, the protocol allows for multiple concurrent services to be taking place at any one time. Services can support other data streams such as video, geopositioning, file transmission, etc.

**Figure 7:**
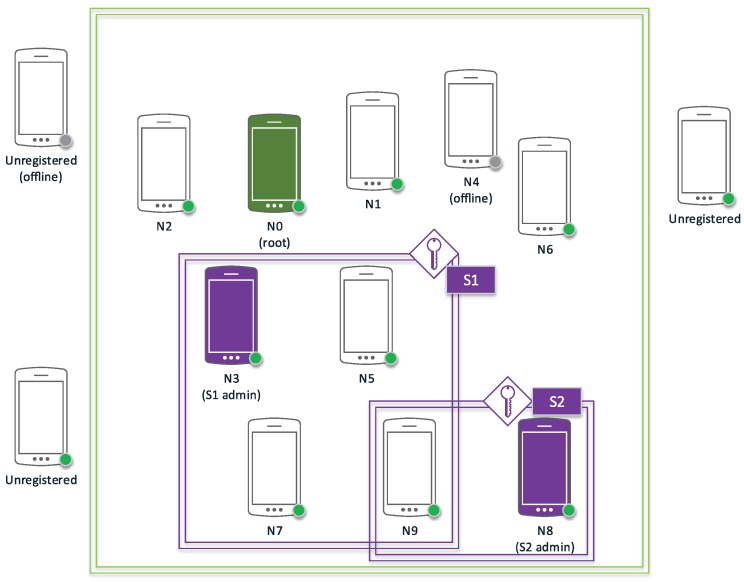
Services (in purple) within a network (in green).

As shown in Figure 7, when a node $N_{i}$ decides to initiate a service *S*, it will need to generate a session key and send it, together with the parameters describing the type of service, to the rest of nodes that will be added to that service. This communication will be performed via a network control message through the secure channel that is available to all network members.

From this moment onwards, node $N_{i}$ is considered an administrator node within *S* and will await the response of all the nodes invited to service *S*. Although most nodes will be added to *S* during the initialization phase, it is possible for $N_{i}$ to add new nodes at a later stage.

**Figure 8:**
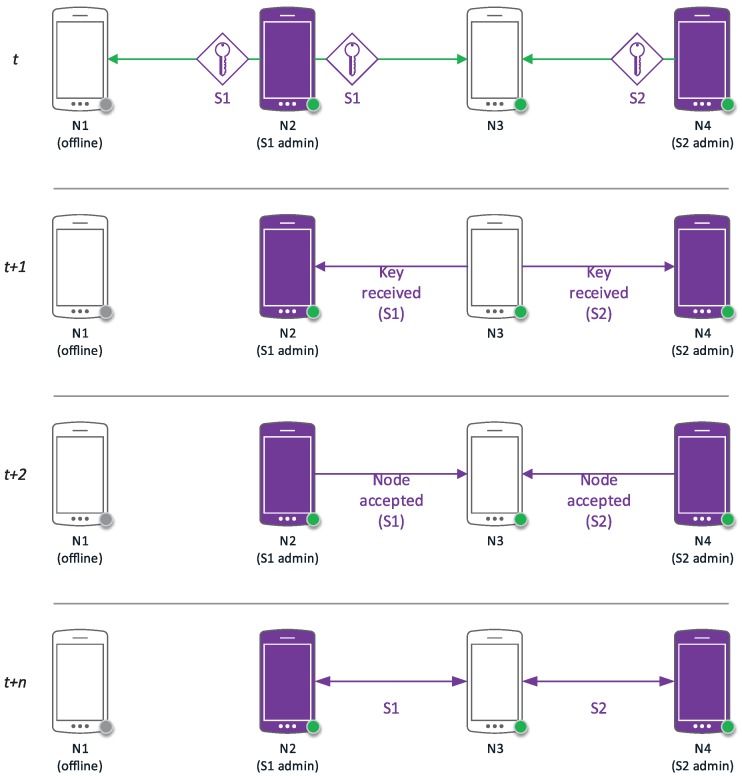
Service initialization chronogram.

In Figure 8, we can see a chronogram representing the creation of two services between four nodes of a five node network ($N_{0},N_{1},N_{2},N_{3},N_{4}$). Both, $N_{2}$ and $N_{4}$ initiate a service; $N_{2}$ transmits the session key of its created service ($S_{1}$) to $N_{1}$ and $N_{3}$; conversely, $N_{4}$ is doing the same with $N_{3}$ and its service ($S_{2}$). After the message exchange regarding admission and information of each service, it can be observed that two services have been created: $S_{1}=N_{2},N_{3}$ and $S_{2}=N_{3},N_{4}$. Node $N_{1}$ has not been able to accept its invitation to service $S_{1}$, since it is offline.

#### 3.3.1. Node Addition

When a node $N_{i}$ is invited by an administrator node to participate in a communication service *S*, it can accept that invitation within the lifetime of the session key by sending a service control message encrypted with the received session key. Then, the administrator will add $N_{i}$ to the repository of nodes pertaining to the service and will propagate this information among all nodes already pertaining to *S*.

#### 3.3.2. Service Topology Management

With the updated local copy of the repository of nodes pertaining to *S*, each node is capable of determining its own representation of the service topology and transmitting the required information accordingly. When a node detects that another node has disconnected or is malfunctioning, whether intentional or due to network conditions, it will send a service control message to the administrator node so that it can consider the exclusion of the malfunctioning node from *S*.

Another important aspect to be considered in the management of a service is that session keys are changed when their lifetime expires. This not only increases cryptanalysis difficulty but also allows to automatically expire unanswered invitations to *S*.

#### 3.3.3. Node Exclusion

A service administrator node can decide, either automatically (depending on configuration parameters) or manually, to exclude a node from the service according to the service control messages that have been received and its own acquired information. In order to do this, the administrator node will delete the excluded node from the repository of nodes pertaining to the service, generate a new session key and propagate it to the remaining nodes in the service. As soon as these nodes receive this information, they will recalculate their topology with the new parameters and act accordingly.

### 3.4. Data Encryption

All packets sent from node $N_{i}$ to a different node $N_{j}$ are encrypted in transit. In order for the data to be decrypted correctly, packets need to have additional unencrypted fields indicating the type of packet and the node identifier which will correspond to the root node for a network message and the service administrator node for a service message; also allowing to choose the correct decryption method depending on the type of message (network or service).

In the implementation described in [7], the type field allows for $2^{4}=16$ different types, with 0 indicating a network message and 1 to 15 indicating a specific service, limiting the total number of simultaneous services to 15. This is adequate for most applications but can be adjusted if a different range is needed.

#### 3.4.1. Authentication

Authentication can be performed employing the Elliptic Curve Digital Signature Algorithm (ECDSA, see [23]) with the FourQ elliptic curve for performance reasons. In this way, every node will have a non-ephemeral key pair with the public key signed by the root node for authentication purposes.

#### 3.4.2. Network Level Encryption

Network packets are always node-to-node communications. Data can be encrypted with a session key that is securely shared via an ECDH based on the FourQ elliptic curve, again for performance reasons. Each node has a set of encryption public/private key pairs for ECDH that is different than the authentication keys. If forward secrecy is desired, these keys can be ephemeral and implementing a double ratcheting algorithm, as described in the Signal protocol (see [3,4,5]). For symmetric encryption, the Advanced Encryption Standard (AES) in Galois/Counter Mode (GCM, see [24]) is an excellent option than can provide data integrity together with encryption. A 128-bit symmetric key is advised (see Table 1) since it is of equivalent security level to the FourQ elliptic curve public key cryptography discussed in this section.

#### 3.4.3. Service Level Encryption

Service packets are encrypted using symmetric encryption with the active session key that has been previously transmitted to those nodes pertaining to the service. This receiving nodes can decrypt the service packets with their stored copy of the active session key for that service. This session key is changed on node exclusion to guarantee that an excluded node will not have access to the active session key and, therefore, will not be able to decrypt any service packet after the exclusion takes place. This is guaranteed even in the case the excluded node (or an attacker) manages to capture the network traffic associated to the service.

The session key is generated by the service initiator and propagated through the service nodes by the means of network level messages. Once this key has been distributed to all service nodes then symmetric data encryption (AES) is used for all service packets.

Forward secrecy can be enabled by forcing a periodic session key change, so that service nodes replace their ephemeral network level key pairs, a new session key is propagated through ECDH and the service is restored.

## 4. Conclusions

In this paper we have analyzed several commonly used and state-of-the-art public-key key-exchange protocols with the aim of establishing the best algorithms for lightweight cryptography in critical infrastructure and emergency scenarios.

With the results obtained, it is very clear that elliptic curve algorithms not only present a certain advantage in terms of key length but, also, a very tangible improvement in overall performance.

These performance improvements are even more significant in critical infrastructure and emergency scenarios where peer-to-peer networks of small wireless devices might be deployed to increase coverage and resiliency to power, cable communication or other standard infrastructure disruptions. In this way, these systems can provide valuable information, communication, and coordination tools like secure messaging, positioning, voice or video conferencing, etc. Therefore, lighter weight cryptography means more efficient use of computing power and battery, which might be a deciding factor.

In those cases where communication protocols implement forward secrecy, it is paramount that key generation is as fast as possible since ephemeral ECDH key pairs are generated per message.

It should also be remarked that, although it is very slow compared to ECDH algorithms, RSA has an advantage for some applications where key pairs are not generated frequently but short values (like secret keys, beacons or other types of granular data) must be encrypted with public-key cryptography.

In most applications, however, FourQ ECDH key exchange would be optimal, especially since the authors have already performed an optimized implementation for ARM processors, which are used by most current mobile device manufacturers. Unfortunately, due to its novelty, it has not been incorporated into common secure protocols yet.

We have also proposed a framework intended to provide security to a previously published real-time audio conferencing protocol based on a peer-to-peer network of nodes in a hypercube topology. This framework builds on the analyzed public-key primitives, including usage of the FourQ ECDH key exchange and ECDSA digital signature algorithms, as well as AES in GCM mode to provide symmetric encryption and data integrity. The proposed framework is, nevertheless, flexible enough to support a wide range of applications.

Detailed implementation and performance analysis of the proposed framework and its adaptation to different applications is planned to be performed in the future. Further analysis of registrar trust and real identity verification schemes for specific applications would also be very interesting.

## Figures and Tables

**Table 1:** Required key length in bits for equivalent security.

Symmetric	RSA/DH	ECDH
80	1024	160
112	2048	224
128	3072	256
192	7680	384
256	15,360	512
